# Zoo Visitors’ Most-Liked Aspects of Elephant Encounters and Related Perceptions of Animals’ Emotions and Welfare States: A Pragmatic Approach

**DOI:** 10.3390/ani14060923

**Published:** 2024-03-17

**Authors:** Angela M. Lacinak

**Affiliations:** Department of Social and Political Sciences, Philosophy, and Anthropology, University of Exeter, St. Luke’s Campus, Heavitree Road, Exeter EX1 2LU, UK; am932@exeter.ac.uk

**Keywords:** elephants, visitors, animal experiences, animal training, animal emotions, animal welfare, zoos, pragmatism, animal–caregiver interactions

## Abstract

**Simple Summary:**

Zoos are home to a diverse range of species, including those in need of conservation. Given the high costs involved in caring for their residents and satisfying their commitments to educating the public, zoos rely on patronage from visitors. Zoos, therefore, need to understand how visitors think and feel about the well-being of the animals in their collections. If visitors perceive zoos as spaces where animals are unhappy, they will be less likely to visit. This qualitative study documented and assessed visitor experiences of and responses to training activities provided for the resident herd of African elephants at Zoo Tampa, Florida, USA. Surveys were conducted with visitors following their attendance at elephant training and enrichment sessions, and they focused on ascertaining visitor perceptions of elephant emotions, care, and welfare. These surveys revealed that visitors perceived that the elephants involved in the encounters were happy, content, calm, comfortable, engaged, excited, good, playful, and safe (among other emotional descriptors) and that they experienced good welfare. Responses also gave insight into why visitors felt this was the case and identified aspects that visitors did not like or that they felt could be improved. The research provides zoos with insight into the impact that animal–caregiver interactions can have on visitors’ perceptions, and it offers a model that could be applied across the sector.

**Abstract:**

Zoos strive to provide excellent welfare for resident animals, including those belonging to endangered species involved in captive breeding programs while addressing visitors’ concerns regarding the ethics of captivity. Zoos also rely on income and support from visitors to provide exceptional care for their animal residents. It is essential, therefore, that zoos consider how visitors perceive their animals’ well-being, including physical and psychological welfare. This pragmatic, qualitative research explores the aspects of training-focused enrichment activities provided for a resident herd of African elephants (*Loxodonta africana*) that visitors liked/disliked, and it assesses perceptions of the animals’ emotions and welfare. Data were collected via surveys following live encounters at Zoo Tampa, Florida (USA). Reflexive Thematic Analysis generated key themes related to visitors’ most/least-liked aspects of the encounters, including proximity, elephant–caregiver relationships (ECRs), educational content, and teaching/learning opportunities for the elephants themselves. Participants in the research perceived the elephants as happy, content, calm, comfortable, engaged, excited, good, playful, and safe (among other emotional descriptors) and as having excellent welfare. Expressions of dislike referred to visitors’ inability to have contact with the elephants, such as via touching or feeding. While focused on one case-study zoo and a charismatic species, this study nonetheless provides zoos with insight into the impact that animal–caregiver interactions can have on visitors’ perceptions, and it offers a model that could be applied to other species and at other zoological institutions. These visitor experiences and visitor perceptions of animal welfare drive future visitor actions, including support for zoos.

## 1. Introduction

In addition to their important roles as sites of conservation, zoos are of value to the visitors who attend them for education and recreation, as evidenced by their strong global patronage [1,2]. Zoos are not without their challenges, however, particularly in relation to public perceptions of the ethics of captivity for charismatic and socially complex animals such as elephants (*Loxodonta africana* and *Elephas maximus*). As an example of this, in Zoo Ethics: The Challenges of Compassionate Conservation, Gray discusses the letters that Melbourne Zoo in Australia receives from visitors concerned with the welfare of their elephants [1]. In her view, these written apprehensions reflect the public’s growing consternation with zoos’ ability to meet the needs of large cognitive and socially complex animals such as elephants [1]. Gray further urges, “to remain relevant and ethically defensible there must be a commitment to respect the interest of each animal held by a zoo, including interest in positive welfare, life and choice” [1] (p. 6).

A specific aspect of zoo elephants’ lives that gives them decision-making opportunities and strives to improve their psychological well-being is positive reinforcement-based learning sessions (referred to colloquially and hereafter as PRT [positive reinforcement training] sessions). In PRT sessions, animals are free to engage or not with their human caregivers and to perform or decline to perform requested behaviors without fear of aversive consequences [3]. This has been documented by Wilson et al. to result in latency regarding behavioral compliance but increased welfare [3]. The choice to participate requires relationships between the elephants and caregivers so that the elephants want to interact. Whitham and Wielebnowski, who call for increased academic and pragmatic attention to zoo animals’ affective states, emphasize the indispensable role that caregivers’ relationships play in the lives of zoo animals and their welfare [4].

As zoos strive to provide excellent welfare for their individual residents while addressing the concerns of their visitors, it is essential to delve beyond the caregivers’ and elephants’ experiences of their interactions and consider how patrons perceive those interactions. Visitor satisfaction is paramount for zoos, as it directly impacts their economic viability, which then affects zoos’ capacity to fund conservation efforts, provide stimulating environments for their resident animals, and employ skilled staff, among other community contributions [2]. Furthermore, this research explores both consumer satisfaction (visitors’ likes/dislikes) with an organizational product (e.g., tours that require a separate purchase from front-gate admission) and animal satisfaction (expressed through visitors’ perceptions of elephants’ emotions and welfare states that are then viewed through the lens of an established welfare assessment tool). These components draw together data at the crux of Zoo Tampa’s and the Association of Zoos and Aquariums’ (AZA’s) missions, both of which reference animal care and visitor engagement [5,6].

### 1.1. Pragmatism

Pragmatism, as an academic epistemology, is based on practical or pragmatic conceptions of problems encountered in life. John Dewey [7], one of the scholars credited with the development of pragmatism, believed that scientific inquiry, influenced by the researcher’s past experiences, should resolve queries with action that brings about change [8]. Pragmatism is not a new research paradigm, though it has resurged, particularly in the social sciences, in recent years (e.g., [8,9,10]).

Kelly and Cordeiro argue that pragmatism is well suited for studies that focus on organizational processes, particularly within non-governmental organizations (NGOs) [11]. They adopted a Deweyan approach to pragmatic inquiry within their own research, centralizing their arguments around the following points: “(1) an emphasis on actionable knowledge, (2) recognition of the interconnectedness between experience, knowing and acting and (3) a view of inquiry as an experiential process” [11] (p. 3). This approach was particularly well suited to my research since it relied on lived experiences that evolved as consequences of elephant–caregiver interactions and zoo–visitor encounters. Though Zoo Tampa at Lowry Park (henceforth Zoo Tampa or ZT) does not identify as an NGO, there exist parallels between zoos and NGOs. Swaisgood notes the public’s confidence in the educational trustworthiness of zoos regarding wildlife and the natural world and ponders the possibility of zoos defining themselves in the future as “conservation NGO[s] that just happen[s] to hold some animals in captivity as part of [their] mission to connect people to nature” [12] (p. 1339).

Pragmatism, in the existing literature, is often utilized in terms of thinking about ethics. For example, Learmonth posited that combining three established ethical frameworks (Compassionate Conservation, Conservation Welfare, and Duty of Care) provides an excellent tool for evaluating the ethics of human–animal interactions (HAIs) in zoos [13]. In this example, Learmonth acknowledges that HAIs can be either beneficial or harmful to humans and other-than-human animals (henceforth animals), identifying a problem that is then acted upon in the form of scientific investigation, resulting in a pragmatic solution that can be implemented at zoos to ensure ethical interactions between the visiting public and resident animals [13]. Indeed, many research inquiries within zoos incorporate some level of pragmatism, as they seek to improve a specific, unexplored aspect of animal care, welfare, visitor experience, etc., that can be improved. In another relevant example, Carlstead et al. explored keeper–elephant relationships in zoos, finding that they benefited the welfare of both beings and provided actionable information for improving the management of elephants and the training of caregivers [14]. Both examples (those of Learmonth and Carlstead et al.) incorporated pragmatic underpinnings to zoo problems with actionable solutions. This is justified, as decision-making in zoos involves pragmatic reflection. However, neither article fully engaged with pragmatism as a philosophical concept. Kupper and De Cock Buning argue for both pragmatism and pluralism in animal ethics discourse [15]. Like Kelly and Cordeiro [11], Kupper and De Cock Buning recommend a Deweyan philosophical pragmatism that embraces a less rigid consideration of the multitude of factors influencing lived experiences, and they caution against a tendency in research to seek an over-simplified, monistic approach to humans’ relationships with animals [15]. This lack of philosophical pragmatism in the zoo ethics and welfare literature deserves reconsideration, as pragmatism is a good lens for understanding areas of deficiency and converting them into opportunities for improvement. Further, it could serve as a model for future visitor engagement studies.

One of the criticisms of pragmatism, as it could relate to this research, is that persons in a dominant societal position, such as administration at a zoo, might resist the contemplation of “hegemonic power structures as problematic and worthy of inquiry” [8] (p. 7). In other words, since humans are the primary decision-makers within zoos, it is feasible that they may not see the need to consider the animals’ perspectives or how the research queries benefit them. However, the recent requirement for AZA-accredited zoos to evaluate the welfare of each animal resident (as opposed to herds or troops, for example) indicates a concern for the welfare (physiological and psychological) of individuals [16]. Rose and Riley, who argue for more structured zoological animal welfare analysis, concur, noting the significant improvements made in animal well-being studies and evaluations over the past two decades [17]. As evidence of US zoos’ commitment to this direction, animal welfare is one of the three tenets identified in the AZA’s mission statement [5]. Still, I attempted to remedy this potential concern by incorporating perceptions of animals’ emotions and welfare states as a component of this study’s findings, placing emphasis on the benefits of the research to both the humans and animals who make up the zoo community.

### 1.2. Aims

This study aimed to explore zoo visitors’ (1) most- and least-liked aspects of African Elephant (*Loxodonta africana*) Backstage (AEB) encounters and (2) perceptions of elephants’ associated emotions and welfare states; then, the study sought to (3) develop actionable next steps for zoo elephant encounters and further research, following a pragmatist epistemology.

## 2. Materials and Methods

The data for this qualitative study were collected in person following live elephant encounters at Zoo Tampa from 13 March to 19 May 2019. During this time, AEB encounters were conducted several times per week, with a zoo-imposed limit of one encounter per day and a capacity of 14 or fewer participants per encounter (yielding a total of 94 participants). Paper surveys comprised of open-ended questions and a Likert-type scale were utilized. The first two days of data collection served as a trial period. During this phase, all the surveys were administered by the researcher to assess the time needed to answer the queries and ensure clarity and ease of interpretation. No one requested clarification, and most of the surveys were completed in approximately five minutes. Post-test surveys were administered by ZT caregivers or interns. Since there were no required changes, and the results of the test phase were congruent with the remainder of the surveys, all viable surveys (including the test phase) were included.

The surveys were limited to one page in order to avoid survey fatigue and maximize participation, following Hacker and Miller, who studied visitors’ perceptions, intent, and attitudes following an observation of elephants on exhibit in a zoo [18]. A brief, scripted explanation of the study was read to the visitors, along with an assurance that participation was voluntary and that individuals would not be identified. The survey included open-ended questions that allowed the participants the freedom to proffer their own descriptors and phrases, as opposed to selection from a pre-determined list. The prompts comprised the following: (1) Please list the one word that you believe best describes the emotional state of the elephant(s) you met today during the demonstration. In other words, how do you believe the elephant(s) felt during the demonstration? (2) What did you like most about the elephant demonstration? And (3) what did you like least about the elephant demonstration? As the question pertaining to the emotional states of the elephants specified “one word”, any descriptors listed following the initial emotion descriptor were excluded.

The survey also included a Likert-type question asking the participants to rate, on a one-to-ten scale where 1 = very poor and 10 = excellent, the welfare of the elephants they met. The question did not identify particular elephants, as the caregivers selected the elephant participants for each encounter based on the social dynamics of the herd, the elephants’ consent (agreeing to shift into particular areas for the interaction), and other management factors. Consequently, there was no predetermined schedule for which or how many elephants would participate (the elephants participated either singly or in pairs; the herd consisted of two juveniles and their mothers). Therefore, the welfare rating represented either one or two elephants encountered but not necessarily each individual. The scale was selected instead of an odd-numbered scale to force visitors to rate the elephants’ welfare; there was no neutral response option. Lastly, a brief demographics section was included.

### 2.1. Sampling

Surveys were offered to AEB encounter participants eighteen years of age and older, employing a form of convenience or availability sampling frequently used in zoo tourism research (e.g., [19,20,21,22]). Ninety-four viable surveys were completed. The respondents’ ages ranged from the late teens to over sixty. Female participants comprised more than 60% of the respondents, and almost 60% of the respondents had a four-year degree or higher. Just over half attested to visiting ZT at least once annually, and one-third held zoo memberships (annual passes). Almost three-quarters were Florida residents.

### 2.2. Setting

ZT was established in the 1930s, housing a small selection of native Florida animals, and it has grown into one of the most-visited zoological parks in the southeastern US (Zoo Tampa, n.d.). My years of experience consulting at this zoo regarding behavior, and previous research conducted with this specific elephant herd, added deep knowledge of the elephant–caregiver relationships, the zoo’s encounter programs, and the elephant residents. Since this study incorporated my reflexive observations, this historical and embodied knowledge further supported the choice of this field site.

**Figure 1:**
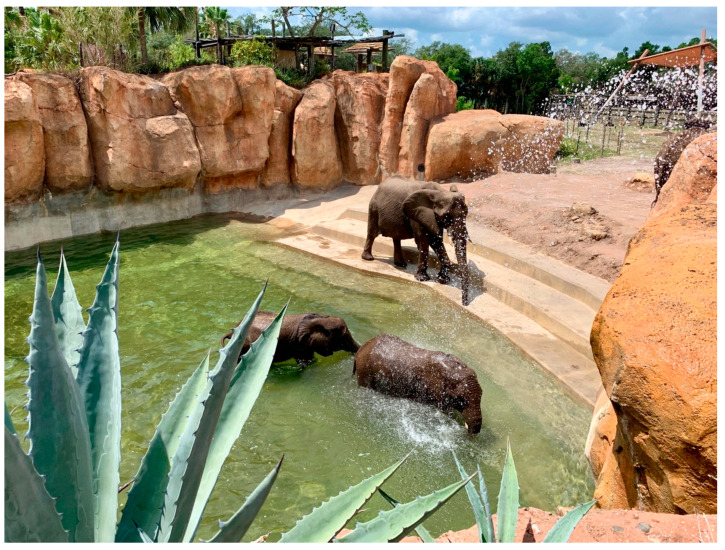
Portion of elephant habitat as viewed from public path. Copyright author.

At the time of this research, AEB encounters were the only opportunity for visitors to experience the zoo’s elephants in an intimate venue. Otherwise, visitors could view the elephants throughout the day in their habitat from a public walking path (Figure 1) or glimpse them in their paddocks from a moving train ride. Likewise, there was no other opportunity to view their PRT, nor to engage with the zoo’s elephant caregivers outside of these private tours. The elephant encounters required an additional fee above the entrance ticket cost.

**Figure 2:**
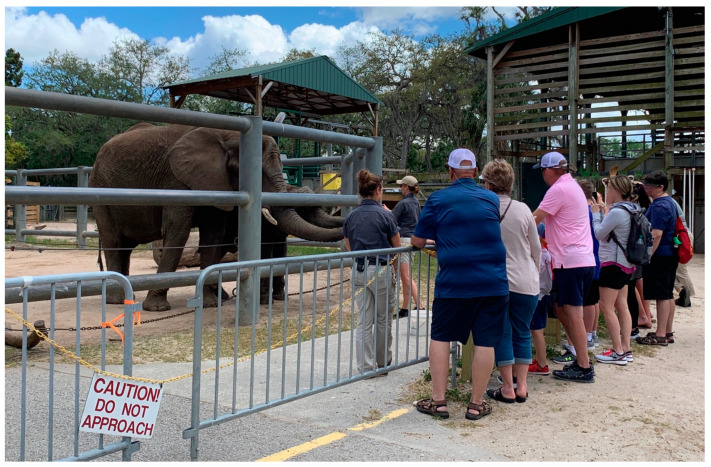
Elephant encounters during data collection. Copyright author.

During the encounters (Figure 2), the visitors were escorted by an educator to the elephant barn, an area inaccessible to general visitors, where they were given a tour of a portion of the elephants’ inside holding space (a large, enclosed, hurricane-grade building), their hay storage room, and two outdoor paddocks. The guests were then introduced to two of the elephant caregivers, who delivered educational content and conducted learning sessions with the elephants (one or two animals). This encounter aligns with Doodson et al.’s definition of “Meet & Greet” offerings in UK zoos, where visitors meet zoo animals under the supervision of staff, often in non-public areas, which may allow visitors more intimate proximity to the animals, with or without direct physical contact or feeding opportunities [23] (p. 11).

All six elephants comprising the herd participated on various days. During the elephant–caregiver interactions (ECIs), the participants were stationed a short distance away from the elephants (behind a barrier), out of reach of their trunks, yet close enough to look into their eyes, speak in a conversational volume to the caregivers, and feel the light breeze created by the elephants as they fanned their ears to cool themselves in the humid, subtropical climate.

### 2.3. Elephant Participants

**Figure 3:**
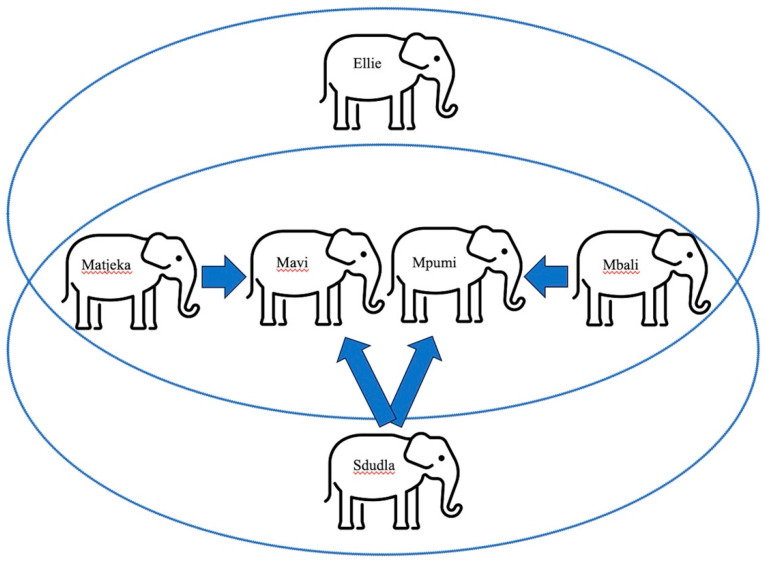
Zoo Tampa elephant social structure. Arrows indicate parentage (i.e., Sdudla is the father of Mavi and Mpumi). Rings indicate permitted full contact (i.e., both mother/daughter pairs interact freely with Ellie and Sdudla on separate occasions). Adapted from [24].

The elephant residents at ZT are one male elephant, Sdudla, and five female elephants: Ellie, Mbali, Matjeka, Mpumi, and Mavi. The elephants’ social interactions are managed in a fission–fusion strategy so that all the animals can interact with others. Not all the ZT elephants can share unrestricted space with one another; Ellie is not allowed to have full contact with Sdudla due to a previous resultant injury, though they have limited access to one another (the ability to touch, smell, hear, and see one another) through bollards. All the elephants participated in the study. Studla and Ellie always participated alone, while mothers/daughters (Mbali/Mpumi and Matjeka/Mavi) typically participated in pairs (though, on one occasion, Mavi participated without Matjeka). Figure 3 illustrates the social structure of ZT’s elephant herd.

### 2.4. Data Analysis

Data generated through the open-ended most/least-liked questions were handled using Reflexive Thematic Analysis (RTA), as described by Braun and Clarke [24,25,26], and following Kelly and Cordeiro’s pragmatic studies [11]. The author’s reflexive observations added further context to enhance the qualitative analysis of the encounters.

## 3. Results and Discussion

### 3.1. Perceptions of Elephants’ Emotions and Welfare States

The elephants were perceived as having positive emotions, as indicated in the descriptors offered regarding how the elephants felt, which were as follows: *beautiful*, *behaved*, *calm*, *comfortable*, *confident*, *content*, *curious*, *engaged*, *enjoying*, *excited*, *exuberant*, *good*, *happy*, *healthy*, *loved*, *motivated*, *natural*, *nice*, *peaceful*, *playful*, *proud*, *relaxed*, *safe*, *satisfied*, and *very good.* Of the descriptors, *happy* was the most-mentioned, offered four times more often (by 44% of participants) than the next most-mentioned emotional descriptor, *content.* The descriptors *calm*, *comfortable*, *engaged*, *excited*, *good*, *playful*, and *safe* received multiple mentions each. All the other descriptors were each listed by only one of the visitors. Since emotions and welfare are intrinsically linked [27,28], it is no surprise that the visitors perceived the “happy” elephants’ welfare as excellent (over 95% rated the elephants’ welfare as 10 on the 1–10 scale; no one rated their welfare below eight).

### 3.2. Most- and Least-Liked Aspects

Four themes were generated, based on the RTA process, regarding visitors’ most- and least-liked aspects of the elephant encounter: the proximity of the elephants to the visitors, the elephant–caregiver relationship (ECR), the educational content, and the teaching/learning of the elephants. Outside of these prominent themes were comments in the least-liked category related to Florida’s intense heat, the unpleasant odor, the duration of the encounter, and the space constraints of the facility, though these mentions were sparse. In fact, it was common for visitors to offer responses to the least-liked aspect query that, from a pragmatist’s view, offered little value regarding needed changes, such as, “N/A”, “nothing”, “liked it all”, and “can’t think of anything”. Many visitors chose to leave this question unanswered. Comments regarding the most-liked aspects of the encounter outside of the four prominent themes were also provided, and they included references to the elephants eating or drinking, the general care of the elephants, the interest that the elephants had in the visitors, and the “ability to be an elephant”. The “ability to be an elephant” comment could reflect the fission–fusion social dynamics of ZT’s elephant herd through which the animals can interact with different elephants on different days. This allows an elephant, like Ellie, who cannot have physical contact with every member of the herd for her own safety, to maintain social bonds with most of the members. On the other hand, the comment could reflect the voluntary nature of the behavior program that allows the elephants the choice to participate or not, or it could reflect other interpretations. The query regarding the most-liked aspect of the encounter also received responses that provided little actionable information, such as “Loved it all!” Below are elaborations on each of the four primary generated themes.

### 3.3. Proximity

The theme of proximity was defined as including comments related to the closeness or intimacy of the encounter. There were mentions of both the most- and least-liked aspects of the encounter regarding proximity. The participants who listed proximity as their most-liked aspect of the encounter expressed this with comments such as “being so up close and personal”, “how close we could get”, and “proximity to elephants”. Contradictorily, some visitors felt that proximity was their least-liked aspect of the encounter, citing their inability to touch or feed the elephants. Examples of specific comments included, “not being allowed to pet them”, “I wanted to feed the elephant: [drawn frown emoticon]”, and “that we couldn’t touch”. One visitor stated, “would have been nice to touch as we did with the rhinos”, referring to the backstage Indian rhinoceros (*Rhinoceros unicornis*) encounter at ZT that does allow physical contact with the animals.

There are valid reasons for this dichotomy of protocols. Elephants’ muscular, dexterous trunks can easily grasp and lift the weight of most humans and are, therefore, a safety concern. This concern is illustrated in the 2023 *Accreditation Standards and Related Policies of the Association of Zoos and Aquariums* (AZA), the largest zoological accrediting body in the US and the organization through which ZT is accredited, which states in regard to caregivers’ safety, “A minimum of two qualified elephant care professionals must be present […] any time an elephant care professional is within trunk’s reach of an elephant” [16] (p. 67). Given this guideline regarding zoo professionals’ interactions with elephants, allowing zoo visitors within trunk’s reach may be risk-prohibitive for the zoo (though visitor interactions with elephants are not prohibited, according to AZA standards [16] (p. 67)).

Zoo visitors have good reason to anticipate opportunities to pet/touch or feed animal residents during encounters. According to D’Cruze et al., who explored animal–visitor interactions (AVIs) in zoos and aquariums with visitors who were either members of or associated with the World Association of Zoos and Aquariums (WAZA), “petting captive wild animals was the most common AVI activity advertised” on facility websites, comprising 43% of all ads [29] (p. 1). Further, AVIs that involved hand-feeding zoo animals (largely hoofed animals, such as giraffes, camels, etc.) were of particular abundance in North America and Oceania, potentially contributing to the expectation of such proximity-related experiences in American zoos [29]. Relatedly, a study of visitor–elephant feeding interactions (where visitors offered browse) was conducted at the Woodland Park Zoo in the US, and concluded that public feedings decreased stereotypic behaviors and increased herd social activity, illustrating that public feedings can benefit both elephants and visitors [30], though it is not a common offering at AZA-accredited zoos.

### 3.4. Elephant–Caregiver Relationships (ECRs)

The theme of ECRs was defined as including references to the interactions between the caregivers and the elephants, to ECRs specifically, and to indicators of affiliative relationships. ECR is purposefully used in lieu of the more traditional HAR (human–animal relationship) to more fully “bring in” the elephant to discourse regarding their lived experiences. The term caregiver is used as an alternative to human or keeper since it better reflects the nature of their work, which shares many similarities with human caregivers [31]. Sample quotes from participants that fell into this theme included the following: “the trust between trainers and elephant”, “seeing demonstrators work so well w/elephant”, “trainer/elephant relationship”, and “they seemed happy to see the trainers”. This finding is supported by a similar study that the current author conducted at the Palm Beach Zoo and Conservation Society involving visitor experiences with Malayan tigers (*Panthera tigris jacksoni*), a Southern ground hornbill (*Bucorvus leadbeateri*), and an American alligator (*Alligator mississippiensis*), for which the animals’ relationships with their caregivers were found to be associated with perceptions of positive welfare and emotional states [28]. As noted previously, the welfare ratings assigned to the elephants were exclusively positive. This limited range precluded the parsing out of positive versus negative welfare ratings against generated themes. However, as is discussed further in this article (see the section on elephants’ [potential] perspective), the Five Domains Model provided indications of positive welfare for ZT’s elephants as perceived by encounter participants [27,28]. There were no comments under this theme heading that were identified as the least-liked aspects of the encounter.

The discourse regarding animal–caregiver interactions, relationships, and bonds was found to be “a relatively recent development” a decade ago [32] (p. 123). More recently, publications have included the exploration of neutral and negative HARs in zoos [33], HABs in zoos [34,35], and HAIs in zoos from a broad ethical perspective [13]. Additionally, HARs with multiple species across taxa have now been studied, e.g., orangutans (*Pongo pygmaeus)* [36], giraffes (*Giraffa camelopardalis*) [37], and a variety of pet reptiles [38]. Most relevant to this research, a 2019 multi-institutional study in the US found that positive ECRs are mutually beneficial and that caregivers are significant in the social lives and well-being of elephants [14].

### 3.5. Educational Content

The theme of educational content was defined as including references to facts or information shared by ZT staff to participants or the general knowledge of the ZT staff. Mentions in this theme were made to conservation, training, and individual elephant history. Some comments were imprecise, such as, “facts given”, “education”, and “the knowledgeable zookeepers”. Others were more descriptive of the educational content that impacted them the most, such as “education of care of elephants”, “the educator explaining [that] the ‘tricks’ have an actual benefit for husbandry & vet care”, and “learning how they [the elephants] got here and about where the $ [sic] at Zoo Tampa is dispensed”. The visitors also listed specific educational facts imparted during the encounter as their least-liked aspect. Examples included the following: “Ellie is blind in 1 eye” and “learning that poaching is still happening”. In these cases, it seemed anecdotally apparent that the educational component of the encounter was not objectionable (i.e., it cannot be said that the visitors did not like receiving the information) but, rather, that the information received was upsetting, surprising, or expressed facts that the visitors wished were not true.

Delivering educational messaging to visitors is a key aspiration of zoological facilities [39]. According to a 2022 study, animal–caregiver encounters (the study focused on cheetahs [*Acinonyx jubatus*]) can result in the retention of new knowledge, increased conservation-mindedness (particularly when the encounter was a component of a guided tour), and positive emotional states for the animals [39]. During the AEB encounters included in the current case study, the caregivers did not recite a written script but typically discussed the phylogenetic history of African elephants, the ontogenetic history of the specific animals that the visitors met, how they taught the elephants behaviors needed to participate in their own care, the importance of those behaviors (frequently for husbandry or medical care), and conservation issues impacting elephants in Africa and Asia. Though there is an assumption that interactions or encounters with zoo animals can increase public education and conservation action, D’Cruze et al. caution that research affirming this assumption is lacking [29]. They, therefore, recommend increased research exploring visitors’ interactions with animals and the subsequent impact on visitors’ actions and perceptions [29]. In this study, the caregivers’ messaging was significant for the visitors, as many of them affirmed it as their most-liked aspect of the encounter.

### 3.6. Teaching/Learning of Elephants

The fourth theme, that of the teaching/learning of elephants, was the most-liked aspect among the largest proportion of participants. The elephant teaching demonstration was but one portion of the AEB encounter, as mentioned previously. Though the teaching demonstrations were included for all the other themes (proximity, ECRs, and educational content), this theme was differentiated since it included comments regarding the teaching sessions/demonstrations and specific behaviors the elephants exhibited that were the result of requests by their caregivers. All responses that fell under this theme were listed as the most-liked aspect; no aspects were listed as the least-liked. As with the other themes discussed, some participants’ answers were vague, such as, “the training”, “the trainer portion”, “the behaviors”, and “seeing how the elephants are trained and worked with”. Again, from a pragmatist’s standpoint, this provides little actionable information. Some visitors, however, recalled specific behaviors as their most-liked aspect, such as when an elephant painted on a canvas or climbed the horizontal bollards with their front legs (standing on their hind legs).

Doodson et al. argue against zoo animals behaving in ways that may be interpreted as unnatural for people’s amusement [23]. Given this, one might assume that requesting elephants to paint or stand on their hind legs should be discouraged. This could be a valid interpretation under conditions of some elephant management programs; however, I argue that the consideration of key details contradicts this assumption. For example, Doodson et al. specify that animals should not be “made to behave”, implying force or coercion [23] (p. 9). As has been discussed regarding ZT’s elephant training standards and philosophies, elephants are given the choice to participate and to engage in specific behaviors. Furthermore, should the elephants find the behaviors of painting or standing on their hind legs against containment-reinforcing, the assumption that the behaviors are unnatural becomes less problematic. Animals, including humans, adapt to their environments and learn to behave in ways that yield desirable outcomes. Additionally, both of the mentioned elephant behaviors demonstrated the natural capabilities of the elephants. Elephants have been widely documented in photos rearing up on their hind legs to forage (an internet search yields hundreds of photos in both wild and captive settings), and the dexterous tips of their trunks have been documented for tool use such as holding branches to scratch their backs [40]. Therefore, these behaviors are natural but are demonstrated in a new context based on their current environments.

Visitors also specifically mentioned when an elephant played with a new toy. Though playing with a toy could be categorized under a different theme of enrichment interaction, in this case, there was an indication that playing with the toy was a behavior being taught to the elephant. This was indicated by comments such as, “playing with his new toy” and “the new toy interaction”. Toys for animals (not just zoo animals) must sometimes be conditioned. As an example, imagine that an elephant had never experienced toy enrichment for mental and physical stimulation. Under this condition, providing him/her with a large elephant-proof ball would have little meaning. Not knowing how to play or interact with the ball without experience and associated consequences would be expected. The caregivers might have to spend time showing the elephant how to interact with the object—pushing it toward the elephant, etc., and then reinforcing appropriate interactions with food, praise, or taction. A history of reinforcement causes the elephant to interact with the ball with increasing frequency. Once toys take on a reinforcing value of their own, they can serve as reinforcers for other behaviors. This functions to expand the reinforcement variety that caregivers can offer to animals, and it simultaneously strengthens the animal–caregiver relationships as the caregivers become associated with an ever-growing variety of positive reinforcers.

### 3.7. Elephants’ (Potential) Perspectives

As zoos strive to assess animal welfare on an individual basis, it becomes requisite to evaluate animals’ behavior for indicators of enjoyment or aversion as they relate to the many elements of their lived experiences. Though elephants’ emotions are internal states, professionals may rely on their experience of the animals’ behavior in other conditions that are likely enjoyable (e.g., play) or fear-inducing (e.g., a lightning strike in proximity). Further, there are established scientific protocols for behavior modification that zoo professionals may reference to predict the future probability of certain reactions. Here, I strove to draw on both my experience in and understanding of the science related to teaching animals and current research on animal welfare assessments to conduct an informed, reflexive analysis of the elephants’ perspectives (see [27,41], discussed further in this text).

Elephants, like humans, are social animals [42]. As previously mentioned, ZT implements a fission–fusion approach to its resident elephants’ herd dynamics to provide a variety of social interactions for each animal. In addition to these conspecific social opportunities, ZT provides formal ACI opportunities for its elephant residents in the form of AEB encounters, organized enrichment activities (with or without humans), and private learning sessions conducted outside of the purview of the visiting public. It also provides numerous daily informal/unplanned interactions that take place when the caregivers speak to, play with, or otherwise engage with the elephants during their daily care activities, some of which are prompted by the elephants themselves.

Though formal interactions with zoo animals are the topic of most of the academic discourse (including this research), they represent only a fraction of the animals’ daily activities. In 2018, I explored this as one aspect of a study involving three elephants from the same herd at ZT [43]. The study, which included 34 learning sessions, found that learning sessions consumed approximately 15 min, or 1%, of each elephant’s day (24 h) [43] (p. 35). When I further reviewed the training records for two months prior to the 2018 study, it was discovered that each elephant was not offered learning sessions daily, reducing the time spent in formal learning sessions with caregivers to 0.5% of their day [43] (p. 36).

Given the deprivation of elephant–caregiver interactive activity and the voluntary nature of the ECIs described in this study, it can be assumed that the elephants find the interactions with their caregivers reinforcing. This assumption follows the premise of reinforcement theory, which contends that the scientific protocols of behavior modification (reinforcers increase behaviors, and punishers decrease behaviors) provide insights into animals’ motivations and emotional states since they either increase their frequency of the behaviors or not [41]. Fennell expounds that “reinforcement theory is thus a powerful mechanism to understand positive and negative emotional states in animals” [41] (p. 327). The behavior of the elephants to consent to participation by approaching their caregivers when requested reflects the anticipation of an expected desirable outcome, rather than the avoidance of an undesirable outcome/consequence.

This assumption is accurate under the management standards of ZT, as caregivers do not share physical space with the elephants, a strategy referred to as protected contact (PC). In PC interactions with ZT elephants, the elephants have the choice to participate or not in learning sessions and, once in session, to refuse specific behavioral requests of their caregivers without repercussions. Further, animals’ environments should provide ample reinforcers that directly compete with the caregivers for interaction (such as food for browsing, enrichment toys, conspecifics, water for play or bathing when appropriate, etc.). This places a responsibility on the caregivers to themselves become highly reinforcing to the animals, to serve as generalized conditioned reinforcers (defined as reinforcers that have been associated or paired with a variety of other reinforcers) [44] (p. 64), and thus increase the likelihood that the animals will prefer the ACI over competing reinforcers in their environment.

Enjoying something, however, is not always an indicator of optimal welfare. Humans, for example, engage in drug use and overeating regardless of the detrimental side effects [41]. Unfortunately, humans are not the only animals to make choices contradictory to positive welfare. This is evident in obese companion and zoo animals; though, in these cases, the food provided is also the responsibility of the caregivers/guardians. Extrapolating this hypothetical to ZT’s elephants suggests it is possible that choosing to engage with their caregivers at the expense of other reinforcers could result in compromised welfare. For example, the caregivers could offer unhealthy foods (many zoo animals have enjoyed marshmallows, gummy candy, sodas, and a wide variety of other nutritionally lacking treats), request behaviors that could harm the elephant (e.g., requesting an animal with an injury or arthritis of the hip to stand on his/her hind legs), or call an elephant away from breeding activities, which could interrupt social bonds or cause frustration and/or aggression. These scenarios are unlikely in modern, accredited American zoos, given that diets are now typically prescribed by nutritionists, the medical care of the animals is closely monitored by a veterinary team, and caregivers are sufficiently aware of the elephants’ estrus cycles and reproductive behaviors so that they would not choose those periods of time for PRT sessions (and if they did, the elephants would likely refuse the sessions, as the opportunity to breed is a primary reinforcer that, at that moment, likely has a higher value than whatever the caregivers have to offer).

A recent tool developed to assess the welfare and associated emotions of animals that includes a parameter involving animal–caregiver interactions is useful in this application [27]. If we consider the emotional descriptors that visitors offered regarding the elephants under the conditions of the encounter, we find that they are associated with positive welfare. In the welfare assessment tool, Mellor et al. provide examples of animal behaviors that contribute to positive affective states such as remaining in close proximity to their caregivers, having a calm demeanor, willingly participating in interactions, seeking contact, etc., many of which were observed by visitors of the ZT elephant encounters [27]. This further affirms the excellent welfare ratings that were selected by participant visitors. Given that the relationships between the caregivers and the elephants were apparent to the visitors, that they perceived the elephants’ emotions as largely happy, and that they rated the elephants’ welfare as positive (a finding supported by an abbreviated application of the Mellor et al. animal welfare assessment tool [27]), it can be said that the elephants probably (though it is acknowledged that feelings are internal to each being and, therefore, cannot be known with certainty) enjoyed the interactions. D’Cruze et al. affirm that well-executed interactions can serve to enrich zoo animals, and conversely, poorly executed interactions can serve to deplete animal welfare [29].

### 3.8. Considerations for Future Elephant Encounters

The pragmatism paradigm requires actionable outcomes based on knowledge acquired. ZT’s AEB encounter participants indicated they were satisfied with their experiences, given the positive comments regarding their most-liked aspects, the dearth of least-liked aspects, their perceptions of elephants’ emotions, and their ratings of elephants’ welfare. As previously discussed, visitors’ engagement through personal encounters with zoo animals has long been an essential component of zoological operations for both pragmatic (funding daily operations) and aspirational (raising funds for conservation work and inspiring an admiration of and respect for nature on a larger scale) reasons. This research indicates that the elephant encounters at ZT can serve to engage and educate their guests while also demonstrating the positive affective and welfare states of the resident elephants.

There remain, however, opportunities for improvement and future inquiries to be explored. Comments referencing the heat or other environmental factors, for example, might be remedied through habitat modification or additional features, such as the installation of shade structures or misting fans. Yet the lack of intimacy or proximity to the elephants was the most-referenced disappointment, and from a pragmatic standpoint, it should be considered for the enhancement of the experience. Therefore, the zoo may wish to consider opportunities that would facilitate visitors’ physical interactions with the elephants without such contact being risk-prohibitive. For example, an elephant could be asked to align himself/herself parallel to the paddock containment and to wrap his/her trunk around a fence post in front of them (a form of targeting or stationing in which an animal orients a particular body part to a particular object). This would put the elephant in a position incompatible with making trunk contact with a visitor. Visitors could then approach the elephant one at a time to touch/pet his/her hind section (with a hand or brush), under the supervision of a caregiver, while a second caregiver focuses on the elephant’s behavior. Alternatively, visitors could make indirect contact with an elephant by reaching a target pole toward the animal with which the elephant could touch the tip of his/her trunk, or visitors could provide water from a hose in which the elephant may choose to play. Many elephants enjoy such activities, particularly on hot summer days (a personal observation). Alternatively, caregivers could proactively educate the visitors about the potential dangers of interacting with wildlife such as a bull elephant in musth.

There are additional options that might satisfy visitors’ desire to have more direct contact with the elephants, such as feeding opportunities. As discussed by Fernandez et al., these interactions can be accomplished safely and with benefits to both animals and visitors [30]. Fernandez et al. found, however, that this may not be an appropriate or equally beneficial activity for every elephant (or visitor), so it would have to be considered on an individual elephant/visitor basis [30]. Furthermore, feeding animals requires the additional management of the nutritional content of visitor-delivered food items to ensure the proper nutrition and calorie consumption of the animals involved. [45]. Sherwen and Hemsworth note the increasing trend of visitor feeding opportunities in zoos and caution that “visitors feeding animals may result in zoo animals associating visitors with a positive experience, but it may also result in mismanagement of nutrition for certain animals, resulting in health problems” [45] (p. 14). Doodson et al. encourage zoos to provide animals with a choice regarding their participation in learning sessions and to ensure that the animals are not made to perform “unnatural” behaviors for visitors’ entertainment, lest they risk being perceived as exploitative and “inadvertently promote[ing] unethical fully-contrived captive-wildlife tourist experiences” [23] (p. 10). Given previous discourse, however, choice-based interactions that the animals find rewarding or enriching can be reasonably incorporated into visitor experiences, even when animals perform behaviors that might seem unnatural, if thoughtful educational messaging accompanies the activity. It is acknowledged that this study represents one elephant herd at one zoo and, therefore, may be limited in its applicability to other zoos, herds, or animal care teams.

Regarding future research inquiries, it is of interest to disentangle the components of the encounters that visitors associate with positive (or negative) elephant emotions and welfare states. Future studies could explore specific reinforcers (i.e., foods, praise, toys, and water play), behaviors (i.e., for veterinary care, exercise, or guest education), and messaging content (i.e., conservation, individual history, natural history, or animal–caregiver relationship details). Also, as full engagement with philosophical pragmatism is rare in the discourse of zoo animal welfare and ethics, opportunities to address real-world problems with pragmatic action resulting from rigorous science abound.

## 4. Conclusions

Published studies to date that evaluate visitors’ perceptions of animals’ emotions and welfare under the context of PRT sessions in zoos are lacking. One such study relates to this research; both explored visitors’ perceptions of animal emotions and welfare states during animal–caregiver interactions, though the previous study did not include elephants, and it used a different data collection methodology [28]. The goal of the current case study was to explore visitors’ most- and least-liked aspects of elephant encounters in a zoo and perceptions of elephants’ associated emotions and welfare states and then to develop recommendations for zoos (which could also be applied to other species and zoos) and future research inquiries. This article speaks to the aim of this special issue by identifying and contributing to the understanding of visitor experiences and perceptions of zoo animal welfare and by setting out how these have considerable potential to influence zoo animal management.

This article has discussed four themes generated using a thematic analysis of visitors’ most- and least-liked aspects of elephant encounters: proximity, ECRs, educational content, and teaching/learning sessions. Most of the comments were positive. When queried regarding their perceptions of the elephants’ emotions (how the elephants felt) based on the AEB experience, “happy” was the most mentioned, and no one described the elephants with descriptors reflecting poor affective states. The visitors’ proffered emotional descriptors and comments were compared to Mellor et al.’s 2020 welfare assessment model, and they were found to reflect positive welfare [27]. Furthermore, most of the visitors directly rated the elephants’ welfare as excellent. This case study suggests that ACIs in zoos, particularly intimate learning sessions, are opportunities to display positive welfare states and related emotions through visible relationships and cognitive stimulation (learning). Further, it provides a dynamic context in which visitors enjoy the dissemination of educational content.

## 5. Limitations

This study is not representative of the general population or of average zoo-goers, as it was centered on zoo visitors who sought to participate in an elephant encounter that required fees in addition to general park admission.

## Data Availability

The data that support the findings of this study are available from the corresponding author upon reasonable request.
